# Development of Chitosan Nanocomposite Films Reinforced with Metal Oxides and Lignin Derivatives for Sustainable Food Packaging

**DOI:** 10.3390/polym18070800

**Published:** 2026-03-26

**Authors:** Ioanna Koumentakou, Petroula Altantsidou, Sofia Stefanidou, Katerina Nikola, Pavlos Efthymiopoulos, Ioannis Tsamesidis, Eleana Kontonasaki, George Z. Kyzas

**Affiliations:** 1 Hephaestus Laboratory, School of Chemistry, Faculty of Sciences, Democritus University of Thrace, 65404 Kavala, Greece; petralta@chem.duth.gr (P.A.); sophiastephanidi@gmail.com (S.S.); aigikol@chem.duth.gr (K.N.); pefthym@chem.duth.gr (P.E.); 2 Department of Prosthodontics, School of Dentistry, Faculty of Health Sciences, Aristotle University of Thessaloniki, 54124 Thessaloniki, Greece; itsamesidis@auth.gr (I.T.); kont@dent.auth.gr (E.K.)

**Keywords:** chitosan, zinc oxide, titanium dioxide, lignin, nanocomposite, food packaging

## Abstract

The development of sustainable packaging materials with advanced functional properties is a key priority for the food industry. In this study, chitosan (CS)-based nanocomposite films incorporating titanium dioxide (TiO_2_), zinc oxide (ZnO), hybrid ZnO_TiO_2_ nanoparticles, lignin (LG), and nanolignin (nLG) were synthesized and comprehensively characterized. Structural analyses (FTIR, XRD, SEM) confirmed strong intermolecular interactions and homogeneous nanoparticle dispersion, particularly for TiO_2_ and low ZnO concentrations. Mechanical testing showed that TiO_2_ and ZnO significantly enhanced tensile strength (up to fourfold) and elongation at break. Among the prepared nanocomposite films, CS-TiO_2_ films at 2 wt% exhibited the best balance of mechanical performance and antioxidant activity. Subsequent incorporation of LG and especially nLG into the CS-TiO_2_ matrix further enhanced flexibility and toughness, antioxidant efficiency, and radical-scavenging activity above 90%, and improved UV-shielding capacity by reducing light transmittance. Moreover, antibacterial testing against *Escherichia coli* demonstrated that CS/TiO_2_/nLG films achieved the highest reduction (~46%), attributed to synergistic electrostatic, oxidative, and phenolic mechanisms. Overall, CS/TiO_2_/nLG nanocomposites emerge as multifunctional, biodegradable films with significant potential for next-generation active food packaging applications.

## 1. Introduction

Current efforts focus on applying nanotechnology to meet consumer demand for food quality assurance, particularly for products with extended shelf life during storage and distribution [1]. Additionally, incorporating antimicrobial ingredients into packaging is being explored to further extend the shelf life of food under such conditions. The food industry must carefully select the most suitable packaging material for each food product, considering the advantages and disadvantages of each option, or incorporate specific features into the packaging material that align with the food’s end-use properties [2].

Beyond the growing demand for sustainable, environmentally safe materials, many recent studies have focused on developing food packaging materials that are biodegradable, rapidly degrade, and completely mineralize in the environment [3]. Among the most promising alternatives are biopolymers, which offer ecological benefits due to their biodegradability. It has also been noted that packaging materials made from biopolymers can be tailored to reduce oxygen and water vapor permeability, lipid transfer among the ingredients of multi-component foodstuffs, and lipid transfer between the environment and foodstuffs [1].

Chitosan (CS) is the second-most abundant polysaccharide after cellulose and is a cationic polysaccharide derived from chitin through partial deacetylation [4]. CS is one of the most widely used biopolymers for packaging owing to its biodegradability, biocompatibility, excellent film-forming capacity, and intrinsic antibacterial properties [5]. Additionally, it has been classified as a safe food preservative in the EU, USA, and China [6]. However, the mechanical and antimicrobial properties of pure CS films are insufficient for practical food packaging applications [7].

In recent years, lignin (LG), a natural polymer and a major by-product of the cellulose industry, has received significant attention in food packaging for its UV resistance, antioxidant activity, hydrophobicity, and biodegradability [3]. However, its poor dispersion and compatibility in polymer matrices remain critical challenges, primarily due to π–π aromatic ring aggregation and hydrogen-bond formation between LG chains. These interactions can result in phase separation and membrane instability, thereby significantly compromising the mechanical integrity of the composite membranes. In their nanosized state, LG nanoparticles (NPs) offer improved morphological, dimensional, and surface chemical stability, thereby mitigating these drawbacks [8]. Despite its beneficial properties, LG incorporation leads to only minor improvements in the mechanical and antibacterial properties of CS films, necessitating the use of additional reinforcing agents to broaden their applications [9].

The incorporation of inorganic nanomaterials, such as nano-SiO_2_ [10], nano-zinc oxide (ZnO) [11], nano-titanium dioxide (TiO_2_) [12], and nano-montmorillonite [13], into biopolymeric matrices has proven an effective strategy for enhancing their mechanical and antibacterial properties. ZnO NPs are environmentally friendly and have attracted significant attention due to their strong antibacterial properties, high stability, photocatalytic activity, and non-toxicity [14]. Furthermore, ZnO-NPs have notable applications in biomedicine, food additives, and catalysis. They exhibit effective antimicrobial activity against a broad spectrum of bacteria, including both Gram-positive and Gram-negative species, and show biocidal effects on bacterial, fungal, and viral organisms [15]. TiO_2_ is a low-cost nanoparticle with high photocatalytic efficiency, chemical stability, and biocompatibility [16]. The addition of TiO_2_ nanocomposite powder has been reported to enhance the mechanical properties of CS-based membranes [17,18].

TiO_2_ also possesses notable antimicrobial activity, as it facilitates photocatalysis under light exposure, producing reactive oxygen species (ROS) such as ·OH, H_2_O_2_, and ·O_2_-, which play critical roles in microbial cell destruction. TiO_2_ photocatalysis has been widely employed to inactivate a broad spectrum of pathogens, including bacteria, fungi, and viruses [7]. However, the photocatalytic antimicrobial activity of TiO_2_ (band gap energy of 3.2 eV) and ZnO (3.3 eV) is limited to activation by ultraviolet (UV) light, specifically at wavelengths below 388 nm and 375 nm, respectively. This constraint is significant because UV radiation accounts for less than 5% of the total solar irradiance that reaches Earth’s surface. To address this limitation, incorporating TiO_2_ and ZnO NPs into CS matrices has been shown to extend their light-absorption capabilities into the visible spectrum, thereby enhancing their practical applicability under ambient light conditions [19].

CS/TiO_2_ composite films supported on cotton fibers have synergistically enhanced antimicrobial properties, demonstrating their utility in photoreactive packaging systems [20]. In parallel, CS/TiO_2_ nanocomposite membranes demonstrated enhanced antioxidant, ethylene-scavenging, antimicrobial, and pH-sensitivity properties, making them promising candidates for smart packaging applications [7]. Another study reports incorporating *Cymbopogon citratus* essential oil and TiO_2_-NPs into CS films for meat packaging, resulting in increased water vapor permeability and tensile strength, indicating enhanced functional performance. Moreover, ZnO was added to the CS/guar gum polymer matrix and used as an edible coating on Ras cheese, effectively inhibiting microbial growth for up to three months [12]. Similarly, films composed of CS, corn starch, ZnO nanoparticles, and glycerol as a plasticizer showed higher antibacterial activity and prolonged preservation than CS/starch films [21]. Furthermore, bionanocomposite films containing CS, carboxymethyl cellulose, and ZnO-NPs exhibited strong antibacterial activity against Gram-positive and Gram-negative bacteria [22]. CS-based films have also been investigated for their effect on the quality of food, such as mangoes, and results showed an extension of shelf life of up to 18 days without any microbial growth or off-flavor [23]. In another study, CS films incorporated with cellulose nanofibers and silver nanoparticles increased the microbial shelf life of bread samples from 5 to 38 days [24]. In addition, Zehra et al. developed CS-based biodegradable films enriched with thyme essential oil, ZnO, polyethylene glycol, nanoclay, and calcium chloride for the packaging of fresh collard greens. These films reduced the water vapor transmission rate, improved tensile strength, retained chlorophyll content, suppressed microbial growth, and preserved the appearance and sensory quality of the vegetables for up to 24 days compared with LDPE and other biodegradable films [25].

The present study incorporated TiO_2_ and ZnO nanoparticles into a CS matrix to prepare CS-based nanocomposite membranes for food packaging applications. Although previous studies have investigated the incorporation of TiO_2_ or ZnO individually within biopolymer matrices, their comparative performance within the same CS system has not been extensively explored. Therefore, this study examines both their individual and synergistic integration (TiO_2__ZnO) into the CS matrix and evaluates the resulting properties. In addition, LG and nano-LG were incorporated into the hybrid nanocomposite films to enhance their UV-blocking capability and antioxidant activity, thereby improving their suitability for food packaging applications. While LG-based additives have previously been incorporated into CS films, their combined use with TiO_2_ nanoparticles within the same CS nanocomposite system has not been widely investigated, to the best of our knowledge. The physicochemical, UV-blocking capacity, mechanical, and antioxidant properties of the developed membranes were comprehensively assessed, and their antimicrobial efficacy was tested against the representative foodborne pathogen *Escherichia coli*, a commonly used Gram-negative model microorganism in food packaging studies due to its frequent association with food contamination and its relatively high resistance to antimicrobial agents [26].

## 2. Materials and Methods

### 2.1. Materials

CS (No. 9012-76-4) with a high molecular weight (310–375 kDa) and a degree of deacetylation >75%, acetic acid ACS reagent (≥99.7%), and LG (No. 8068-05-1) alkali were obtained from Sigma-Aldrich Co. (St. Louis, MO, USA). Sodium hydroxide (NaOH, No. 1310-73-2) was supplied from Chem-Lab NV (Zedelgem, Belgium). ZnO nanopowder (Product no. NG04SO3803, NPs size: 18 nm, 99.5% purity) and TiO_2_ nanopowder (Product no. NG04SO3507, NPs size: 28 nm, >99.5% purity) were supplied by Nanografi Nanotechnology (Ankara, Turkey).

### 2.2. Methods

#### 2.2.1. Manufacture of Hybrid ZnO_TiO_2_ NPs

For the preparation of the combined ZnO_TiO_2_ nanoparticles, the synthesis relied on Wang et al. [27] and Li et al. studies [28]. Equal amounts of ZnO and TiO_2_ were added to 250 mL of deionized water under magnetic stirring, and the mixture was stirred for 1 h. The homogeneous solution was placed in a stainless steel Teflon autoclave for hydrothermal synthesis of ZnO_TiO_2_ NPs. The autoclave was placed in a furnace at 180 °C for 2 h and then removed and left at room temperature for 24 h. The autoclave precipitate was then collected and placed in an oven at 60 °C for 36 h to dry and obtain the hybrid ZnO_TiO_2_ nanoparticles [29,30]. Under these conditions, ZnO nanoparticles nucleate and grow on TiO_2_ surface, forming n-n semiconductor heterojunction, where Zn-O-Ti bonds develop. The latter is further confirmed by XRD analyses, where the final composite exhibits the characteristic peaks of both metal oxides [31].

#### 2.2.2. Synthesis of Lignin Nanoparticles (nLG)

nLG was synthesized based on the Frangville et al. method [32]; 0.6 g of LG alkali powder was dissolved in 100 mL of 1 N KOH solution. The pH of the resulting solution was carefully adjusted to 4 by the dropwise addition of 1 N HCl under vigorous stirring. The suspension was then neutralized by washing 3–4 times with distilled water, followed by centrifugation at 4000 rpm for 15 min at room temperature. The resulting precipitation was collected and resuspended in distilled water to a final volume of 50 mL. The suspension was then subjected to probe ultrasonication at 40% amplitude for 20 min. The resulting nLG dispersion was used for subsequent characterization and film preparation.

#### 2.2.3. Bionanocomposite Films Preparation

To prepare CS-TiO_2_ films a similar procedure to Dong et al. was followed [33]. TiO_2_ was first dispersed in deionized water at final concentrations of 0.5%, 1%, and 2% *w*/*w* relative to CS. These concentrations were selected based on previous literature reports and were chosen to remain compatible with potential food-contact applications [24,34]. The dispersion was subjected to ultrasonic treatment to ensure effective NPs dispersion. Subsequently, CS (1% *w*/*v*) was added under continuous magnetic stirring, followed by the addition of acetic acid to a final concentration of 2% *v*/*v*. The resulting mixture was stirred for 1 h and cast into Petri dishes. Drying was conducted in a conventional oven at 50 °C for 36 h to achieve complete solvent evaporation. The same procedure was followed for preparing CS-ZnO and CS-ZnO_TiO_2_ composite films. Table 1 provides an overview of the film formulations and corresponding abbreviations, indicating the concentrations of CS and the incorporated nanoparticles (NPs).

The LG and nLG were incorporated into the CS-TiO_2_ film containing 2% TiO_2_, as it exhibited the most promising properties for food packaging applications after the characterization and property evaluation. For the fabrication of CS-TiO_2_/LG and CS-TiO_2_/nLG films, a similar method to that of Vijayakumar et al. was followed [35]. Specifically, TiO_2_ (2% *w*/*w* of CS) was dispersed in deionized water and sonicated, followed by the addition of CS (1% *w*/*v*) under magnetic stirring. Acetic acid was then introduced at a final concentration of 2% *v*/*v*, and the mixture was stirred for 1 h. In parallel, under stirring, LG and nLG solutions were prepared by dissolving the respective compounds in an aqueous NaOH solution (6 g/L). The CS-TiO_2_ solution and the LG or nLG solution were combined and stirred until a homogeneous mixture was achieved. The final mix was poured into Petri dishes and dried at 50 °C for 48 h to ensure complete solvent removal. The formulation was adjusted to maintain a CS:LG or CS:nLG mass ratio of 1:100.

#### 2.2.4. Characterization of the Prepared Films

All prepared films were characterized via FTIR analysis. The Perkin–Elmer Frontier, Shelton, CT, USA attenuated total reflectance Fourier transform infrared spectrometer (ATR-FTIR, ZnSe) was used in the lab to perform the FTIR study, with a nominal resolution of 2 cm^−1^. The spectral range was 4000–550 cm^−1^ [36]. A Bruker D8 FOCUS X-ray diffractometer, XRD (Bruker AXS LLC, Madison, WI, USA) fitted with CuKα radiation (λ = 0.154 nm) was utilized to measure crystallinity throughout a 2θ range of 5–70°. A JEOL JSM-6390LV scanning electron microscope, SEM (JEOL Ltd., Tokyo, Japan) was used to describe the morphology of the prepared films. The size of nLG was studied using data provided by NTA instrument NanoSight LM10 (Malvern Panalytical, Malvern, UK).

#### 2.2.5. Antioxidant Activity

The antioxidant activity of the films was evaluated using the 2,2-diphenyl-1-picrylhydrazyl (DPPH) radical-scavenging assay according to the method of Blots (1958) [37] with slight modifications. Briefly, 100 mg of each film sample was cut into small pieces and immersed in 4 mL of a 0.1 mM DPPH solution prepared in methanol [38]. The mixtures were kept in the dark at room temperature for 4 h to allow the diffusion of antioxidant compounds from the film into the DPPH solution. During this process, the reduction in the DPPH radical leads to a color change from violet to pale yellow. After incubation, the absorbance of the solution was measured at 517 nm using a UV–Vis spectrophotometer. The radical-scavenging activity (%) was calculated using Equation (1):

(1) $$DPPH\;radical\text{-}scavenging\;activity=\frac{Absorption\;of\;control-Absorption\;of\;test}{Absorption\;of\;control}\;\times \;100$$

#### 2.2.6. UV-Blocking Properties

The Shimadzu 2550 UV–vis spectrometer, with a wavelength range of 200–800 nm, was used to analyze the UV absorbance of three distinct substances [38].

#### 2.2.7. Mechanical Properties

The thickness of each film was measured using a dial thickness gauge. For each sample, the average thickness was calculated from four measurements taken along the edges and one at the center. Mechanical properties were evaluated using an Instron Universal Testing Machine (Model 8801, Norwood, MA, USA) operated at a crosshead speed of 1.0 mm/min at room temperature (23–27 °C). Test specimens were prepared in a dumbbell shape. Tensile strength (TS) and elongation at break (EB) were reported as the mean values from three replicates [39].

#### 2.2.8. Antibacterial Activity

The antibacterial activity of the prepared materials was evaluated against the aerobic Gram-negative bacterium *Escherichia coli* [40]. The bacterial strain, obtained as a clinical isolate, was identified using standard biochemical tests according to the procedures described by Cowan and Steel. An overnight culture of *E. coli* was prepared in TSY broth (tryptic soy broth supplemented with 0.5% yeast extract) at 37 °C and the bacterial suspension was adjusted to approximately 10^7^ CFU/mL.

The tested materials were suspended in sterile TSY broth at a concentration of 5 mg/mL and sterilized by UV irradiation for 30 min prior to use. The suspensions were then inoculated with 1% (*v*/*v*) of the bacterial culture and incubated at 37 °C in a 5% CO_2_ atmosphere for 24 h. After incubation, bacterial viability was determined using the plate count method. Briefly, aliquots of each culture were serially diluted in TSY broth and appropriate dilutions (10^−6^) were spread onto TSY agar plates (TSY broth containing 1.5% agar). The plates were incubated under the same conditions for 24 h, after which colony-forming units (CFUs) were counted from three independent observers to determine the number of viable bacteria. Cultures grown in TSY broth without the tested materials served as positive controls. All experiments were conducted in triplicate, and the antibacterial activity was expressed as the percentage of bacterial reduction relative to the control group, calculated based on CFU counts, according to the following Equation (2). Ncontrol is the mean CFU count of the control culture, and Ntreated is the mean CFU count of the culture containing the tested materials.

(2) $$Bacterial\;reduction\;(\%)\;=\;\frac{Ncontrol-Ntreated}{Ncontrol}\;\times \;100$$

#### 2.2.9. Statistical Analysis

Statistical analysis was performed using an independent t-test. Differences were considered statistically significant at *p* < 0.05. Different letters indicate statistically significant differences between the samples.

## 3. Results and Discussion

### 3.1. CS-ZnO, CS-TiO_2_ and CS-ZnO_TiO_2_ Nanocomposite Films

#### 3.1.1. Characterization of the Prepared Biofilms

**Figure 1:**
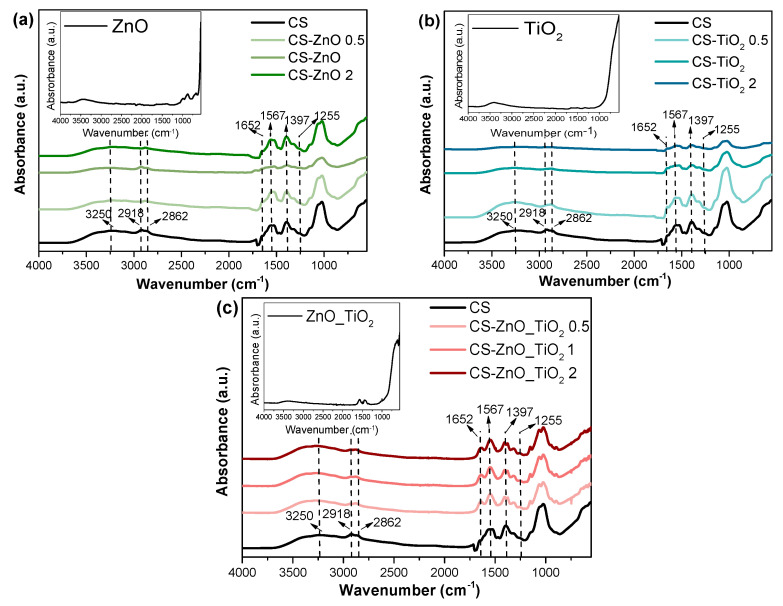
FTIR spectra of (**a**) CS, ΖnO and CS-ZnO, (**b**) CS, TiO_2_ and CS-TiO_2_, and (**c**) CS, ZnO_TiO_2_ and CS-ZnO_TiO_2_ nanocomposite films. The arrows indicate the main characteristic absorption peaks of the samples, and the dotted lines represent reference positions used to compare peak shifts among samples.

The intermolecular interactions among the various constituents in the system were evidenced by FT-IR spectroscopy measurements (Figure 1). A broad band over 3250–3470 cm^−1^ in the pure CS spectrum is due to N–H and O–H stretching. The characteristic bands are approximately at 2862 and 2918 cm^−1^ and can be attributed to C–H asymmetric and symmetric stretching, respectively. The absorption bands at 1652 cm^−1^, 1567 cm^−1^, and 1397 cm^−1^ are assigned to amide I and amide II (C–O stretching), N–H bending vibrations of NH_2_ group, and CH_2_ wagging coupled with OH group originated from CS [41].

FTIR spectra of CS nanocomposites showed that the band at 3250–3470 cm^−1^ shifted to a lower frequency than in CS, because of the electrostatic interaction between CS and NPs (N-H-O---Ti and N-H-O---Zn). CS films with TiO_2_, ZnO, and ZnO_TiO_2_ showed a weak absorption of C-H asymmetric stretching of the methylene group at 2568 cm^−1^ in contrast to CS, which showed a medium intensity, confirming the electrostatic interaction between the hydroxyl groups of CS and Ti^+2^ (O---Ti) as well as Zn^+^ (O---Zn). More intense asymmetric bands of CH_2_ are due to the dipole moment variation produced by electrostatic interaction [1]. O-H deformation vibration appeared at 1255 cm^−1^ as a medium band for CS and a weak band for nanocomposite films, confirming the Ti-O and Zn-O [21]. Additionally, the C-O stretch vibration at 1024 cm^−1^ was strong for CS and became weaker and shifted to a lower frequency in the nanocomposite films due to electrostatic interactions between the C-O group and Ti and Zn. The O-H strong bending vibration around 627 cm^−1^ for CS was shifted to a higher frequency and became weaker in nanocomposite films due to weaker hydrogen bonding [22].

**Figure 2:**
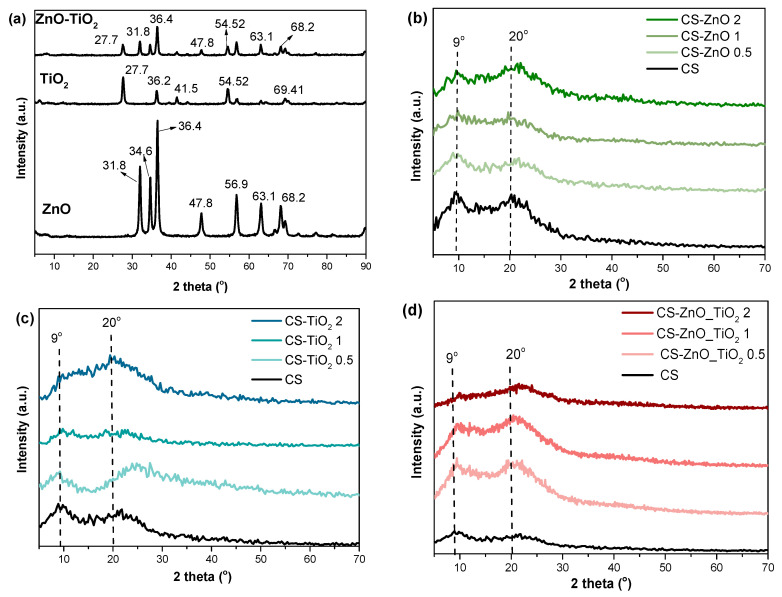
XRD patterns of (**a**) ZnO, TiO_2_, and ZnO_TiO_2_, (**b**) CS, CS-ZnO, (**c**) CS, CS-TiO_2_ and (**d**) CS, CS-ZnO_TiO_2_ nanocomposites containing different ratios of NPs. The arrows indicate the main characteristic absorption peaks of the samples, and the dotted lines represent reference positions used to compare peak shifts among samples.

Figure 2 exhibits the XRD of nanocomposites containing different ratios of ZnO, TiO_2_, and ZnO_TiO_2_. ZnO-NPs have firm diffraction peaks at 2θ  =  31.8° (100), 34.6° (002), 36.4° (101), 47.8° (102), 56.9° (103), 63.1°, and (112) 68.2°, confirming their crystallinity behavior [21]. The XRD analysis of TiO_2_ exhibited prominent peaks at 2θ = 27.7°, 36.2°, 41.5°, 54.52°, 56.95°, and 69.41°, corresponding to anatase (101), rutile (110), anatase (101), and anatase (200) crystal phases, respectively. Additionally, the XRD pattern of hybrid ZnO_TiO_2_ illustrates all characteristic peaks of ZnO and TiO_2_-NPs. Moreover, the XRD profile of the pure CS sheet showed characteristic peaks at 9° and 20° (2θ), which are typical fingerprints of CS semi-crystallinity.

All bionanocomposite films generally exhibited comparable XRD profiles (Figure 2b–d). As shown in Figure 2b, CS exhibited a semi-crystalline structure, with characteristic diffraction peaks at 2θ~9° and 20°, which correspond to the (020) and (110) planes of the CS crystalline lattice. In the nanocomposite films, the XRD profiles maintained these characteristic peaks but with reduced intensity and increased broadening, suggesting a decrease in the degree of crystallinity due to the incorporation of the additives into the CS matrix [42,43]. This broadening of peaks in the nanocomposites suggests the formation of hydrogen bonds between the CS matrix and the incorporated ZnO, TiO_2_, and ZnO_TiO_2_ -NPs, which interfere with the regular packing of polymer chains and increase the amorphous content of the films, thereby reducing their overall crystallinity [19].

**Figure 3:**
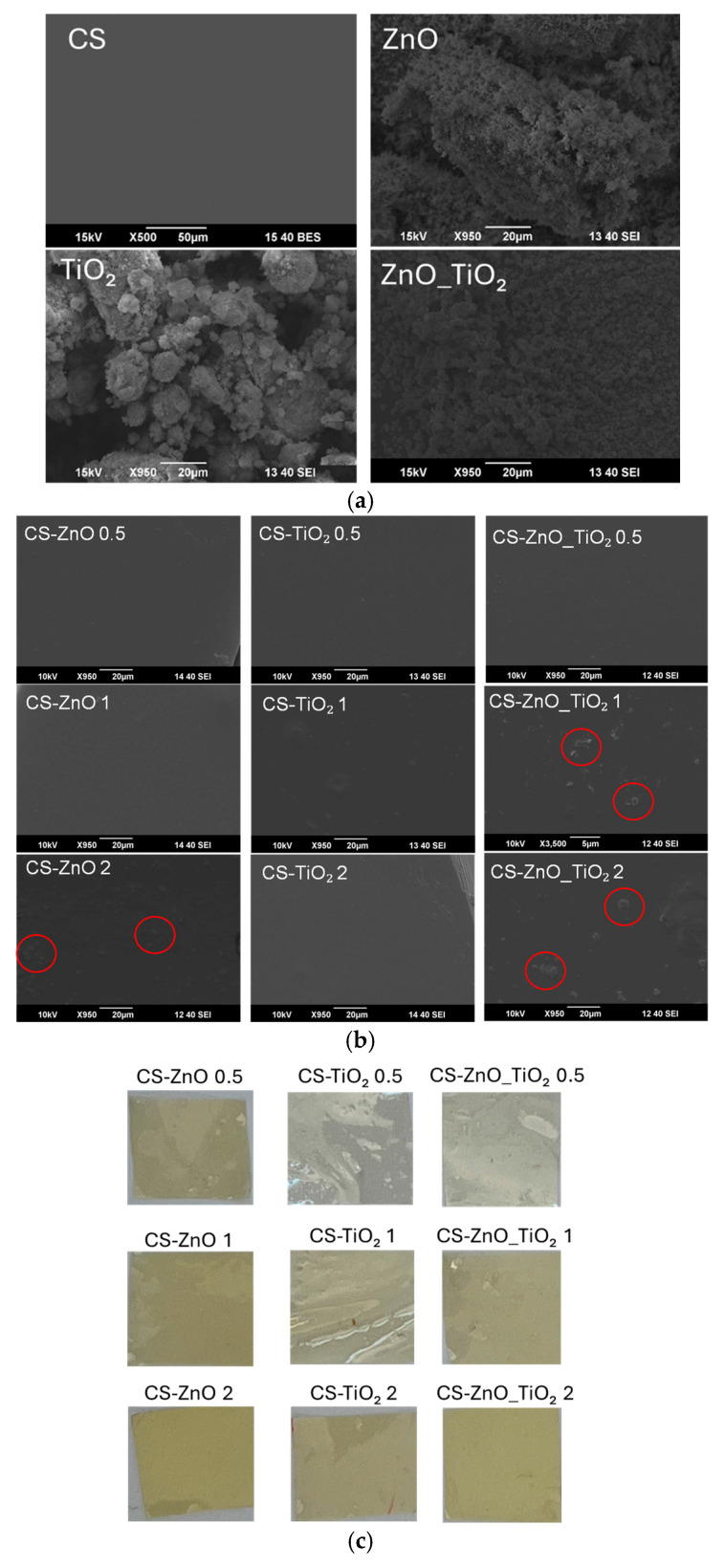
SEM images of (**a**) CS, ZnO, TiO_2_, ZnO_TiO_2_, (**b**) CS-ZnO, CS-TiO_2_ and CS-ZnO_TiO_2_, red circles indicate the presence of nanoparticles distributed on the film surface, (**c**) opacity of the film samples.

The surface morphology of the synthesized nanoparticles ZnO_TiO_2_ and nanocomposite films was examined using SEM, as illustrated in Figure 3. The SEM images of pure ZnO, TiO_2_, and ZnO_TiO_2_ hybrid nanoparticles revealed distinct morphological characteristics. ZnO nanoparticles exhibited a predominantly rod-like or hexagonal structure, whereas TiO_2_ particles showed a more spherical and irregular morphology [44]. The ZnO_TiO_2_ hybrid nanoparticles demonstrated a mixed or intermediate morphology, combining features from both metal oxides, indicating successful formation of a composite structure at the nanoscale [45]. As illustrated in Figure 3a, the surface of the pure CS film appeared smooth, compact, and homogeneous. In contrast, the incorporation of nanoparticles led to distinct morphological variations in the nanocomposite films. SEM analysis of CS-based films containing ZnO, TiO_2_, and ZnO_TiO_2_ nanoparticles showed apparent differences in surface structure. Films containing lower concentrations of ZnO (CS–ZnO 0.5, CS–ZnO 1) and all TiO_2_-based composites (0.5–2%) maintained a continuous, uniform, and relatively smooth morphology, suggesting good nanoparticle dispersion within the polymer matrix. These results corroborate the XRD findings of a robust interaction between the CS and ZnO and TiO_2_ [46]. However, films with higher ZnO loading (CS–ZnO 2) and all CS-ZnO_TiO_2_ nanocomposites exhibited heterogeneous surfaces characterized by nanoparticle agglomeration and non-uniform distribution [47]. Such aggregation likely arises from increased particle–particle interactions at higher filler content and reduced polymer–particle compatibility, which influences the films’ microstructural integrity [48,49,50].

Moreover, opacity is essential for controlling light transmission through food products, as it directly influences their appearance and protection against light-induced deterioration. As shown in Figure 3c, the films’ opacity increased with nanoparticle concentration, suggesting a denser polymer network. CS-based films containing 0.5% nanoparticles exhibited a slight yellow coloration, and the color intensity increased with nanoparticle concentration [51].

#### 3.1.2. Antioxidant Activity of the Prepared Biofilms

Assessing the antioxidant activity of food packaging films is essential, as oxidative processes can lead to spoilage, discoloration, and nutrient loss in food products [33]. Incorporating antioxidant functionality into packaging helps inhibit free radical formation, extending shelf life and maintaining food quality [52].

**Figure 4:**
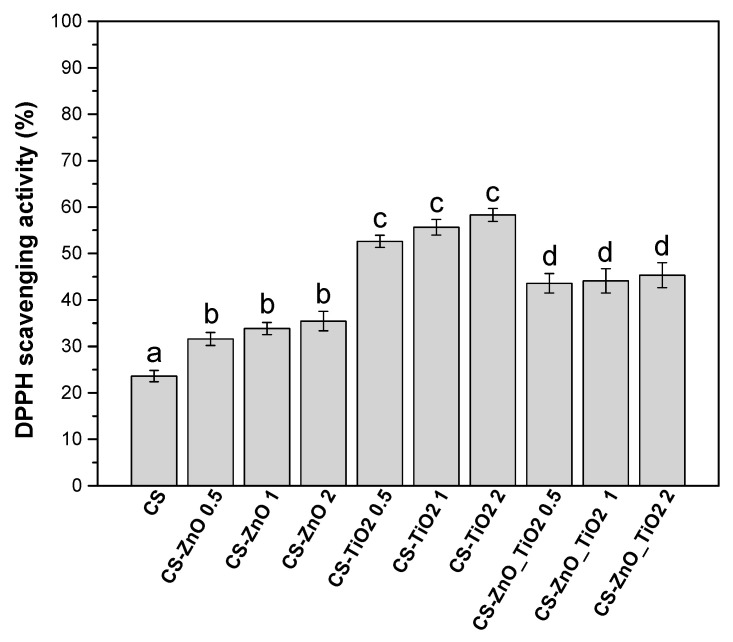
Antioxidant activity of CS and nanocomposite films; different letters suggest statistically significant differences (*p* < 0.001) among groups. Error bars represent the standard deviation of measurements.

The antioxidant activity of the CS-based nanocomposite films was evaluated using their free radical-scavenging capacity in the DPPH assay. The characteristic absorbance of DPPH at 517 nm decreased upon interaction with the nanocomposite films, indicating successful scavenging of DPPH free radicals [53]. This reduction in absorbance was directly correlated with the increasing weight percentage of nanoparticles incorporated into the polymeric matrix, as shown in Figure 4 [54]. The enhancement in antioxidant activity can be attributed to the intrinsic chemical properties of the nanomaterials, which influence their ability to transfer electrons to neutralize free radicals [55].

CS exhibited an antioxidant capacity of 23.6% due to the presence of free amino and hydroxyl groups, which can act as electron donors [56]. However, this activity was significantly enhanced upon incorporating metal oxide nanoparticles.

Among the formulations, films containing TiO_2_ nanoparticles exhibited the highest antioxidant activity, reaching 58.32% at 2 wt%. This effect is due to the high surface area of nanoparticles and the efficacy in scavenging free radicals using DPPH [57]. This trend aligns with findings from a previous study, showing the reliability of the results [58,59]. The CS-ZnO_TiO_2_ hybrid films demonstrated intermediate antioxidant activity (up to 45.36%), suggesting a synergistic interaction between ZnO and TiO_2_, albeit not as potent as TiO_2_ alone. This may be due to partial competition between the two oxides when combined, leading to less available surface area per particle type for radical interaction [60].

On the other hand, films containing ZnO nanoparticles showed the lowest enhancement among the nanoparticle-containing systems, with antioxidant activity increasing from 31.6% to 35.46% as nanoparticle content increased [61]. The enhanced antioxidant activity of CS-TiO_2_ films highlights the potential of TiO_2_ as an effective additive for active food packaging applications where antioxidant ability is essential. Statistical analysis confirmed that the differences in antioxidant activity among the formulations were statistically significant (*p* < 0.05), as indicated by the different letters above the bars in Figure 4.

#### 3.1.3. Mechanical Properties

The applicability of any film as a packaging material is strongly influenced by its mechanical properties, particularly TS and EB. As shown in Table 2, the incorporation of nanoparticles led to an increase in film thickness across all nanocomposite films compared to pure CS (0.055 mm). The addition of ZnO nanoparticles significantly improved the TS of the films, with TS increasing from 12.84 ± 0.62 MPa in pure CS to a maximum of 42.35 ± 0.78 MPa in CS-ZnO films. This improvement can be attributed to the strong intermolecular interactions and cross-linking effect generated between ZnO nanoparticles and polymer chains [62]. However, when the ZnO content was increased to 2 wt%, the TS decreased to 25.67 ± 0.49 MPa, suggesting that excessive loading led to nanoparticle agglomeration, as shown in SEM images [49]. EB decreased sharply from 30.11 ± 0.63% in pure CS to 12.31 ± 0.44% in CS-ZnO 2 films, indicating reduced flexibility. However, the CS-ZnO 1 sample displayed a high EB value of 33.39 ± 0.76%, higher than the control and other ZnO formulations, suggesting an optimal nanoparticle–polymer interaction that improved ductility [63]. The literature supports these findings. For instance, Md. Momtazur Rahman et al. incorporated ZnO nanoparticles into a PLA/CS polymer matrix and observed that the nanoparticles acted as nano-reinforcements, facilitating strain transfer between the matrix and the filler. However, tensile strength (TS) decreased as the ZnO nanoparticle content was increased to 3 wt% [64].

The incorporation of TiO_2_ nanoparticles also enhanced the mechanical performance of the films. TS increased progressively with TiO_2_ concentration, reaching a maximum of 49.86 ± 0.47 MPa at 2 wt%, while EB increased to 34.99 ± 0.24%. These results indicate that TiO_2_ nanoparticles acted as effective reinforcing fillers, enhancing cohesion forces within the polymer matrix and contributing to a more uniform network, consistent with previous reports [65]. SEM observations further supported these findings, showing improved homogeneity in TiO_2_-containing films. These results are consistent with similar research, which reported that the TS and EB of the CS film were enhanced, thereby increasing the TiO_2_ nanoparticle content [66].

In contrast, films containing ZnO_TiO_2_ exhibited reduced mechanical properties compared to single-filler systems. TS decreased from 22.65 ± 0.36 MPa at low concentrations to 15.42 ± 0.42 MPa at 2 wt%, while EB dropped from 22.63 ± 0.41% to 18.74 ± 0.26%. This decline can be explained by the aggregation of mixed nanoparticles within the polymer matrix, which likely disrupted the uniform structure and limited stress transfer, as confirmed by SEM analysis [67].

### 3.2. LG and nLG-Loaded CS-TiO_2_ Films

The CS-TiO_2_ 2 formulation was selected for its high antioxidant and mechanical performance, incorporating both LG and nLG. For consistency and clarity in the presentation of subsequent results, the CS-TiO_2_ 2 film, hereafter referred to as CS-TiO_2_, is retained as a reference point to facilitate direct comparisons with its counterparts containing LG and nLG.

#### 3.2.1. Characterization of the CS-TiO_2_/LG and CS-TiO_2_/nLG Prepared Biofilms

**Figure 5:**
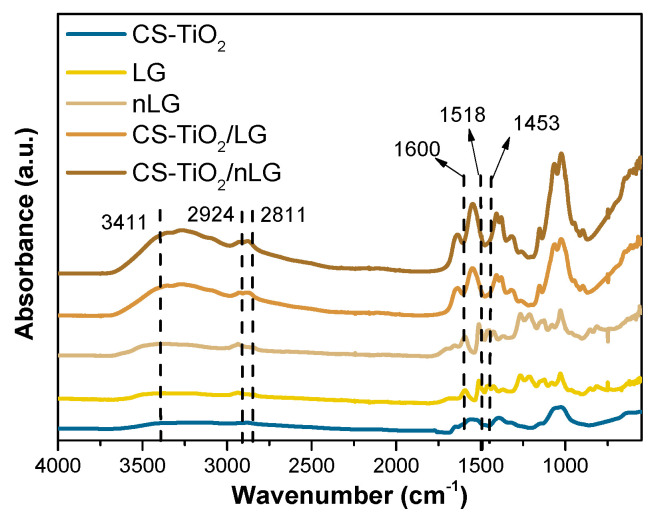
FTIR spectra of CS-TiO_2_, LG, nLG, CS-TiO_2/_LG and CS-TiO_2_/nLG. The arrows indicate the main characteristic absorption peaks of the samples, whereas the dotted lines represent reference positions used to compare peak shifts among samples.

Figure 5 presents the FTIR spectra of the CS-TiO_2_, LG, nLG, and the synthesized composite films CS-TiO_2_/LG and CS-TiO_2_/nLG. Initially, LG and nLG have spectra that are practically identical. A wide band at 3411 cm^−1^ is assigned to aromatic and aliphatic O–H groups. The absorption bands in the region between 2924 and 2811 cm^−1^ were attributed to stretching of C–H bonds in the -OCH_3_ groups of LG. In addition, the intense bands at 1600, 1518, and 1453 cm^−1^ correspond to the typical aromatic ring vibrations of the phenylpropane skeleton [38].

The band corresponding to -OH groups at 3411 cm^−1^ increased significantly in the CS-TiO_2_/LG and CS-TiO_2_/nLG spectra due to the introduction of CS and TiO_2_ with abundant hydroxyl groups [68]. CS-TiO_2_/LG and CS-TiO_2_/nLG films showed the characteristic peaks of both LG and CS. The peak at 1652 cm^−1^ (bending of the N-H of the primary amine) and at 1567 cm^−1^ and 1527 cm^−1^ may have disappeared as a result of interactions between the amine group of CS and the aromatic ring of LG. The CS-TiO_2_ spectra showed shifts in the peaks owing to C-O stretching from 1154 cm^−1^ to a less intense peak at 1149 cm^−1^ in the composite. This suggests that the functional groups of CS (amino, hydroxyl, and carbonyl groups) and LG (hydroxyl, carbonyl, etc.) may have formed hydrogen bonds [69].

**Figure 6:**
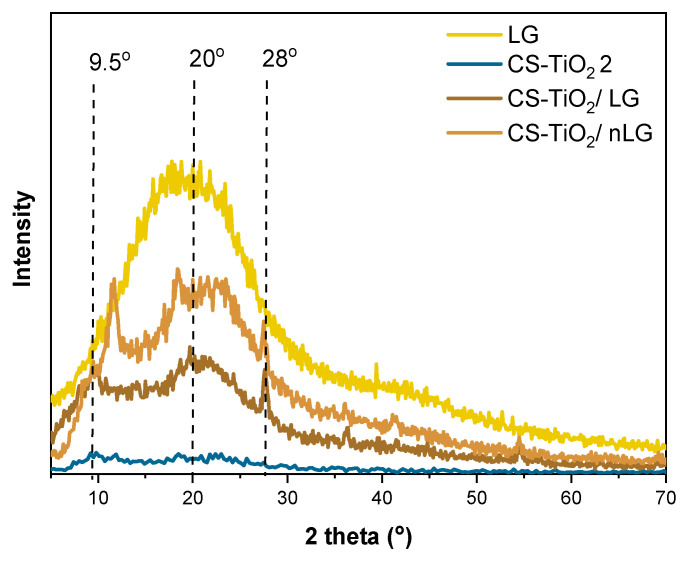
XRD patterns of CS-TiO_2_, LG, CS-TiO_2_/LG and CS-TiO_2_/nLG.

The XRD pattern of LG (Figure 6) exhibits a broad peak centered at approximately 20°, consistent with its predominantly amorphous character. In the CS–TiO_2_/LG and CS–TiO_2_/nLG nanocomposite films, two broad peaks are observed at 2θ ≈ 9° and 20°, corresponding to the semi-crystalline domains of CS. In contrast, an additional peak at 2θ ≈ 28° confirms the incorporation of TiO_2_. Notably, the relative intensities of these characteristic diffraction peaks differ between composites containing LG and those incorporating nLG, indicating that the introduction of LG into the CS film matrix induces changes in the crystallinity of the materials, which has been reported in similar study [38].

**Figure 7:**
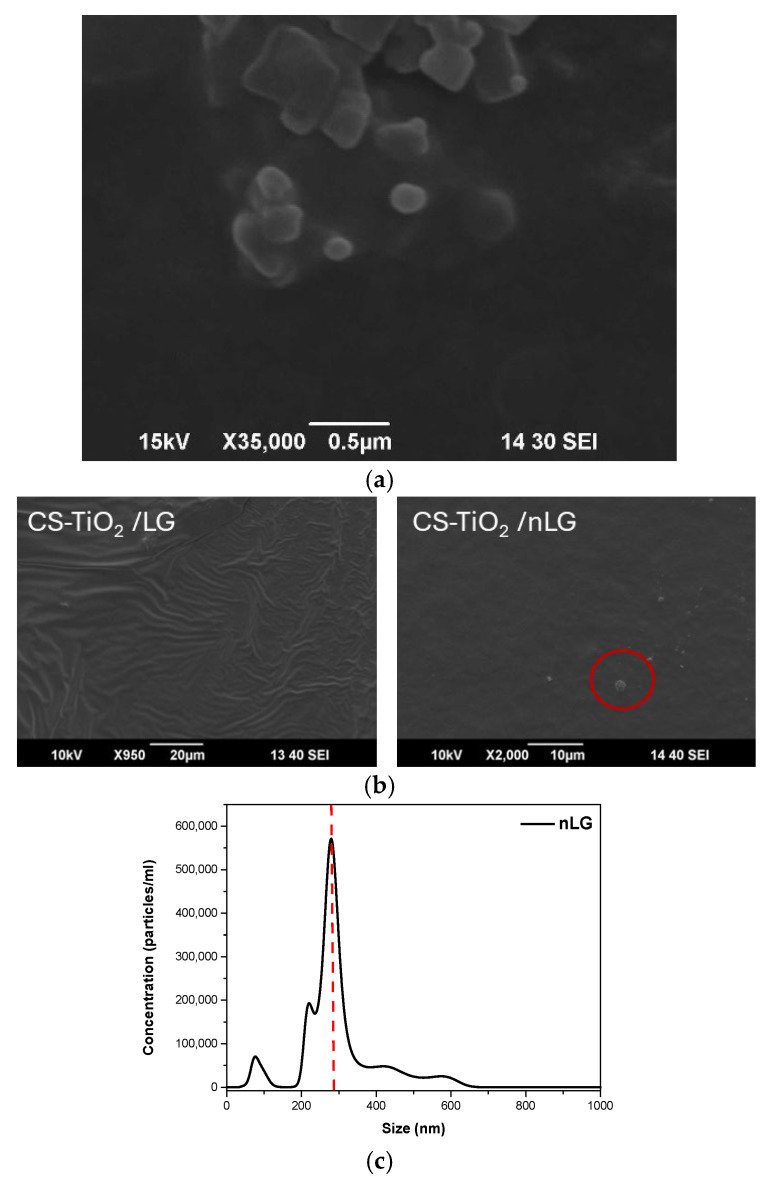
SEM images of (**a**) nLG, (**b**) CS-TiO_2_, CS-TiO_2_/LG, and CS-TiO_2_/nLG films, red circles indicate the presence of nanoparticles distributed on the film surface, and (**c**) the size analysis of nLG, the red dotted line indicates the mean particle size.

Figure 7a confirms the successful synthesis of nLG nanoparticles, which exhibit a predominantly spherical morphology with an average diameter of 278 nm (Figure 7c) [70]. The nanoparticles appear well-defined and uniformly sized, suggesting a controlled fabrication process and minimal agglomeration. This morphology is consistent with previous studies on nanoscale LG structures, highlighting their potential for stable dispersion within polymeric matrices [71].

The surface morphologies of the CS–TiO_2_, CS–TiO_2_/LG, and CS–TiO_2_/nLG films were examined using SEM, as presented in Figure 7b. All films exhibited relatively smooth and homogeneous surfaces, with no visible pores, cracks, or agglomerations, indicating good film-forming ability and uniform dispersion of components within the CS matrix. The absence of phase separation or roughness suggests effective interfacial compatibility among CS, TiO_2_, and LG-based additives [6,50]. However, the CS–TiO_2_/nLG films displayed discrete spherical nanoparticles on the film surface (highlighted in red circles in Figure 7b), which are attributed to the nLG nanoparticles. These spherical features confirm the preserved morphology of the nLG during film casting and suggest partial migration of nLG to the surface during solvent evaporation. This surface presence contributes to enhanced functional properties such as UV shielding and antioxidant activity [38].

#### 3.2.2. Antioxidant Activity

**Figure 8:**
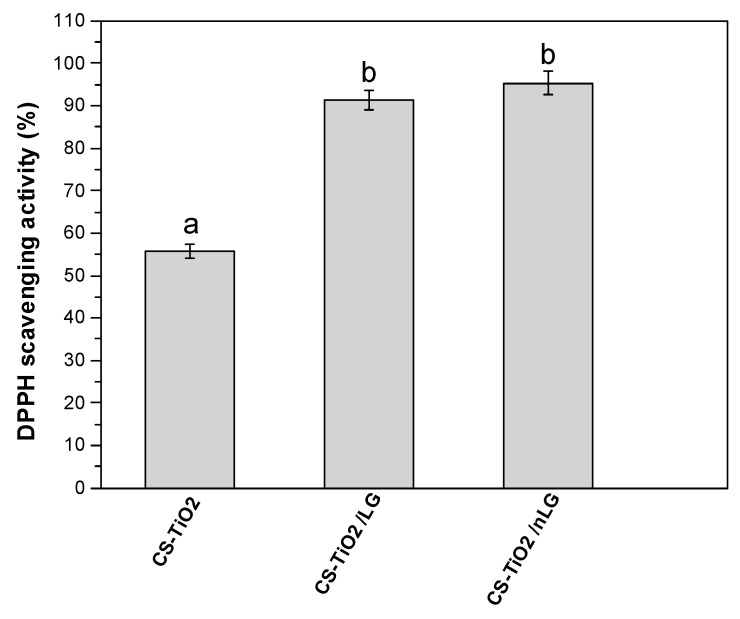
Antioxidant activity of CS-TiO_2_, CS-TiO_2_/LG, and CS-TiO_2_/nLG films; different letters suggest statistically significant differences (*p* < 0.001) among groups. Error bars represent the standard deviation of measurements.

The antioxidant capacity of the CS-TiO_2_-based biofilms was significantly enhanced by the incorporation of LG and nLG, as evidenced by the DPPH radical-scavenging assay (Figure 8). Incorporating LG, especially nLG, into the polymer matrix significantly improved free radical-scavenging efficiency. Specifically, adding LG increased the antioxidant activity to 91.23%, while using nLG further elevated it to 95.36%. This substantial enhancement is attributed to the high antioxidant properties of LG, which donates hydrogen from Phe-OH to DPPH molecules. Then, the Phe-OH radical is successfully stabilized by the OMe group, demonstrating the beneficial effect of OMe on LG’s high antioxidant activity [72]. Furthermore, by interacting with aryl radicals and creating electron pairs, DPPH free radical molecules can achieve stability [73]. The improved antioxidant performance of nLG compared to LG can be attributed to its higher surface-to-volume ratio, which provides more active sites for radical scavenging. Additionally, the nano-dimension of nLG promotes better integration into the CS-TiO_2_ matrix, enhancing the film’s homogeneity and facilitating more uniform surface exposure of antioxidant groups [74].

Compared to previously reported studies, the antioxidant performance of the LG-enhanced biofilms observed in this work aligns well with established literature [38]. However, the antioxidant activity exhibited by the CS-TiO_2_/nLG films in this study exceeds most values reported for LG nanoparticles incorporated into comparable polymeric matrices [75]. This enhanced performance underscores the efficacy of the nanoparticle synthesis and dispersion strategy employed, which likely facilitated improved interfacial compatibility, increased active surface area, and more efficient radical neutralization within the biofilm network. Statistical analysis also confirmed significant differences among the CS-TiO_2_, CS-TiO_2_/LG, and CS-TiO_2_/nLG films (*p* < 0.05), as indicated by the different letters in Figure 8.

#### 3.2.3. UV-Blocking Property

**Figure 9:**
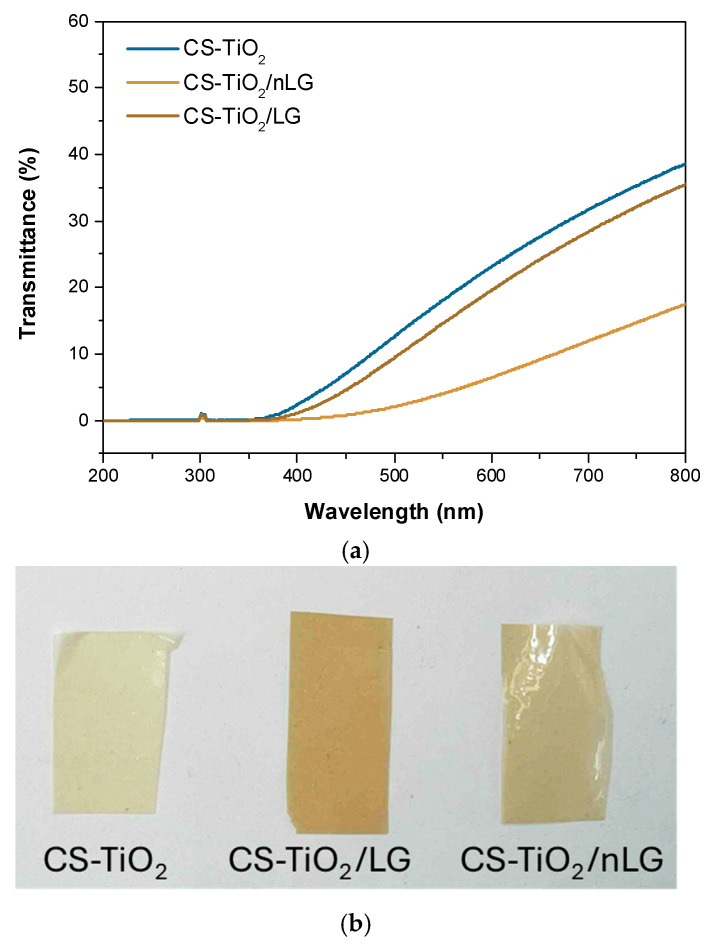
(**a**) UV transmittance of film samples with the wavelength in a range of 200–800 nm and (**b**) opacity of the film samples.

Measuring UV-blocking capacity is crucial for food packaging films, as ultraviolet radiation can accelerate lipid oxidation, nutrient degradation, and microbial growth, thereby shortening shelf life. Effective UV shielding helps preserve food quality, safety, and appearance during storage and distribution [76]. Figure 9a presents the UV–Vis transmittance spectra of the developed films over the 200–800 nm wavelength range. As expected, incorporating LG into the CS-TiO_2_ matrix significantly reduced light transmittance, especially in the UV region [74]. The electrical structure of nano-TiO_2_, which has the ability to absorb light with an energy of hv that equals or surpasses its band gap energy, is responsible for the UV-blocking mechanism of CS-TiO_2_. The UV-ray portion of the solar spectrum contains the band gap energy of TiO_2_. However, the addition of LG and nLG in CS-TiO_2_ exhibited increased UV-blocking capacity, with the CS-TiO_2_/nLG film demonstrating the most pronounced UV-shielding behavior [77].

Both CS-TiO_2_/LG and CS-TiO_2_/nLG films showed a sharp decline in transmittance at approximately 350 nm, effectively blocking harmful UVC (100–280 nm) and UVB (280–315 nm) radiation. This enhanced UV barrier performance is primarily attributed to the high content of phenolic and chromophoric structures in LG, which efficiently absorb UV radiation [77]. The carbonyl group is formed when LG is exposed to UV radiation due to electron transfer, making it easier to extinguish UV light [78]. The film thus effectively protects against UV radiation by converting the photon energy from this source into heat energy or re-emitting it at a lower energy [38].

Interestingly, although the CS-TiO_2_/nLG films exhibited reduced visible light transmittance compared to the CS-TiO_2_/LG films, they demonstrated higher opacity (Figure 9b). This suggests that incorporating nLG not only enhanced the UV-blocking efficiency but also improved visual uniformity, which may enhance consumer appeal and suitability for food packaging applications. As confirmed by SEM, the spherical morphology and smaller particle size of nLG promote more uniform distribution within the polymeric matrix, enhancing interfacial interactions and maximizing UV-absorbing surface area [74].

#### 3.2.4. Mechanical Properties

The mechanical performance of the prepared hydrogel films was significantly influenced by the incorporation of LG and its nanostructured form [79]. As shown in Table 3, the tensile strength was increased to 52.66 ± 0.33 MPa upon adding LG (CS/TiO_2_/LG). A further enhancement was observed when nLG was introduced (CS/TiO_2_/nLG), with the tensile strength reaching 55.41 ± 0.36 MPa. This improvement can be attributed to the effective stress transfer at the polymer–LG interface, where salt bridges between the protonated amino groups of CS and the carboxyl groups of LG contribute to forming a stable crosslinked framework [80]. The nanostructured LG particles provide a larger surface area and better dispersion within the polymer matrix, further strengthening the polymer network and enhancing load-bearing capacity [81].

The EB values also showed a substantial increase with LG incorporation. While CS/TiO_2_ exhibited an EB of 34.99 ± 0.24% (Table 2), the value increased to 48.63 ± 0.28% for CS/TiO_2_/LG and 59.75 ± 0.32% for CS/TiO_2_/nLG. This suggests that LG, especially in its nanoscale form, imparts greater flexibility and ductility to the film matrix [82]. All samples can be ductile since a film is considered brittle when EB is below 5% [83]. Notably, the presence of LG reinforces the film and mitigates brittleness by enabling more uniform stress distribution and greater energy dissipation during deformation. The thickness of the films remained relatively constant across the samples (0.075–0.078 mm), indicating that the observed differences in mechanical properties are primarily due to the structural interactions at the molecular level rather than dimensional variations [84].

Conventional synthetic non-biodegradable polymers such as high-, low-, and linear low-density polyethylene (HDPE, LDPE, and LLDPE), polypropylene (PP), and polyethylene terephthalate (PET) are among the most widely used materials in the modern food packaging industry due to their favorable mechanical properties and durability [85]. Polyethylene (PE) typically exhibits tensile strength values ranging from approximately 9 to 28 MPa, with LDPE presenting values of about 9–15 MPa and HDPE 21–28 MPa [86]. PP generally shows tensile strength values of 40–50 MPa [87], while PET can reach tensile strengths of approximately 55–75 MPa, with elongation at break values often exceeding 100%, depending on processing conditions and polymer grade [88]. In contrast, biopolymer-based films such as CS, starch, cellulose and gelatin generally present tensile strength values between 5 and 50 MPa, depending on formulation and plasticizer content [85]. The incorporation of nanofillers has been widely reported to enhance the mechanical performance of CS films. For instance, TiO_2_-reinforced CS nanocomposites have demonstrated tensile strength values of approximately 18–35 MPa due to improved interfacial interactions and reinforcement effects within the polymer matrix [17]. Similarly, LG-based fillers have been reported to enhance both the strength and flexibility of CS films by promoting hydrogen bonding and improving stress transfer within the polymer network. For instance, the incorporation of 2.5 wt% LG and lactic acid into CS films resulted in a tensile strength of approximately 30 MPa and an elongation at break of 60% [89]. In this context, the tensile strength and elongation at break values obtained in the present study (up to 55.41 MPa and 59.75%, respectively) are comparable to or higher than many previously reported CS-based nanocomposite films and approach the mechanical performance required for certain conventional food packaging applications.

### 3.3. Antibacterial Properties

**Figure 10:**
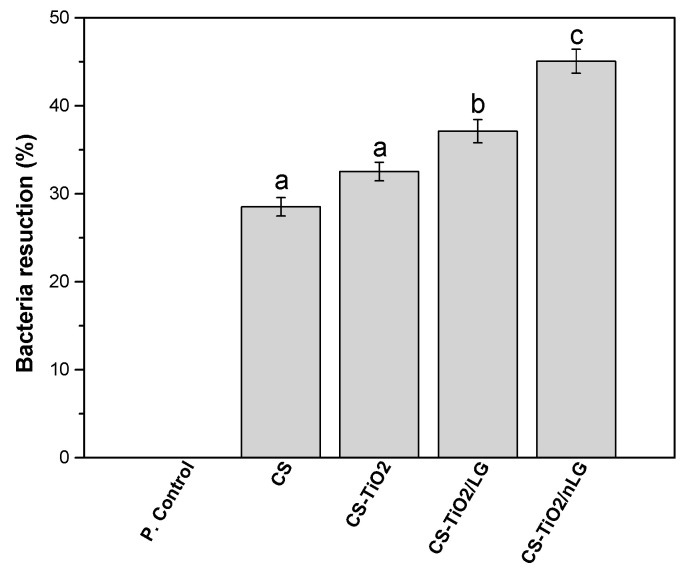
Antibacterial activity of prepared biofilms against *E. coli*; different letters suggest statistically significant differences (*p* < 0.001) among groups.

The results are shown in Figure 10, demonstrating that CS exhibited a bacterial reduction of approximately 30%, confirming its intrinsic antibacterial activity. This effect could be attributed to the electrostatic interaction between the positively charged amino groups of CS and the negatively charged bacterial cell membrane (*E. coli*), leading to membrane disruption and leakage of intracellular components. The incorporation of TiO_2_ slightly enhanced the antibacterial effect (32%), without however statistically significant difference. Comparable findings were also presented by Liangfan Qu et al., who prepared CS/TiO_2_ films with antibacterial properties against *E. coli* by 21.78% [17]. A more pronounced and statistically significant effect was observed when LG was introduced, with bacterial reduction increasing to ~38%. This enhancement is related to the presence of active functional groups in LG, such as hydroxyl (–OH), carbonyl (C=O), and carboxyl (–COOH), which contribute to antibacterial interactions [90]. In agreement, MAS Ali et al [91] reported that phenolic compounds in LG influence the antibacterial mechanism, either by disrupting the bacterial cell membrane and releasing intracellular contents, or through the action of monophenolic compounds such as cinnamaldehyde, which can penetrate bacterial cells, lower intracellular pH, and deplete adenosine triphosphate (ATP) reserves. The CS/TiO_2_/nLG composite exhibited the highest antibacterial efficiency, achieving ~46% bacterial reduction. The antibacterial performance of this composite is attributed to the high surface area and greater accessibility of functional groups, thereby amplifying the antibacterial action of the CS-TiO_2_ matrix [91]. Moreover, it has been reported that nanomaterials can penetrate microbial membranes due to their small size, leading to alterations in membrane integrity and function. Once inside the cell, they can interfere with metabolic pathways, inhibit enzyme activity, alter gene expression, and contribute to protein deactivation, further enhancing their antibacterial efficacy [92].

## 4. Conclusions

This work successfully developed multifunctional chitosan (CS)-based nanocomposite films incorporating TiO_2_, ZnO, hybrid ZnO_TiO_2_ nanoparticles, and lignin derivatives (LG and nLG) for sustainable food packaging applications. The integration of these organic and inorganic fillers effectively overcame the inherent limitations of pure CS films, including poor mechanical strength, moderate antioxidant capacity, and limited antibacterial activity. Among the formulations, CS-TiO_2_ films containing 2 wt% TiO_2_ exhibited the most balanced performance, significantly improving mechanical and antioxidant properties compared with ZnO- and ZnO_TiO_2_-based films. Further reinforcement with LG, especially nLG, led to a substantial enhancement in functional activity. The presence of phenolic and oxygen-containing functional groups in LG, combined with the nanoscale dimensions and large surface area of nLG, contributed to enhanced free radical-scavenging capacity (>90% antioxidant efficiency) and markedly enhanced UV-shielding ability. These characteristics are crucial for preventing oxidative degradation and photo-induced spoilage of packaged food. The films also displayed notable antibacterial activity against *Escherichia coli*. While CS alone reduced bacterial viability by ~30%, the synergistic effects of TiO_2_ and nLG maximized antibacterial efficiency to ~46%. This highlights the potential of CS/TiO_2_/nLG films as active materials that can extend the microbiological safety and shelf life of food products, and it also contributes to the broader goal of reducing plastic waste and advancing sustainable packaging technologies.

## Figures and Tables

**Table 1:** Abbreviations of prepared films and their contents of CS and NPs.

Sample	CS (% *w*/*v*)	ZnO (% *w*/*w*)	TiO_2_ (% *w*/*w*)	ZnO_TiO_2_ (% *w*/*w*)	LG (% *w*/*w*)	nLG (% *w*/*w*)
CS-ZnO 0.5	1	0.5				
CS-ZnO 1	1	1				
CS-ZnO 2	1	2				
CS-TiO_2_ 0.5	1		0.5			
CS-TiO_2_ 1	1		1			
CS-TiO_2_ 2	1		2			
CS-ZnO_TiO_2_ 0.5	1			0.5		
CS-ZnO_TiO_2_ 1	1			1		
CS-ZnO_TiO_2_ 2	1			2		
CS-TiO_2_/LG	1		2		1	
CS-TiO_2_/nLG	1		2			1

**Table 2:** Mechanical properties of films.

Sample	Tensile Strength (MPa)	Elongation at Break (%)	Thickness (mm)
CS	12.84 ± 0.62	30.11 ± 0.63	0.055 ± 0.002
CS-ZnO 0.5	29.69 ± 0.34	21.71 ± 0.23	0.061 ± 0.003
CS-ZnO 1	42.35 ± 0.78	33.39 ± 0.76	0.067 ± 0.001
CS-ZnO 2	25.67± 0.49	12.31 ± 0.44	0.072 ± 0.002
CS-TiO_2_ 0.5	32.65 ± 0.43	30.63 ± 0.36	0.063 ± 0.002
CS-TiO_2_ 1	41.74 ± 0.39	32.25 ± 0.71	0.069 ± 0.001
CS-TiO_2_ 2	49.86 ± 0.47	34.99 ± 0.24	0.075 ± 0.003
CS-ZnO_TiO_2_ 0.5	22.65 ± 0.36	22.63 ± 0.41	0.063 ± 0.004
CS-ZnO_TiO_2_ 1	18.36 ± 0.71	20.15 ± 0.45	0.070 ± 0.002
CS-ZnO_TiO_2_ 2	15.42 ± 0.42	18.74 ± 0.26	0.076 ± 0.001

**Table 3:** Mechanical properties of CS-TiO_2_/LG and CS-TiO_2_/nLG films.

Sample	Tensile Strength (MPa)	Elongation at Break (%)	Thickness (mm)
CS-TiO_2_/LG	52.66 ± 0.33	48.63 ± 0.28	0.078 ± 0.002
CS-TiO_2_/nLG	55.41 ± 0.36	59.75 ± 0.32	0.076 ± 0.003

## Data Availability

The original contributions presented in this study are included in the article. Further inquiries can be directed to the corresponding authors.
